# Genotype-Informed Versus Empiric Management Of VirEmia (GIVE MOVE): study protocol of an open-label randomised clinical trial in children and adolescents living with HIV in Lesotho and Tanzania

**DOI:** 10.1186/s12879-020-05491-9

**Published:** 2020-10-19

**Authors:** Jennifer Anne Brown, Isaac Ringera, Ezekiel Luoga, Molisana Cheleboi, Namvua Kimera, Josephine Muhairwe, Buntshi Paulin Kayembe, Mosa Molapo Hlasoa, Lorraine Kabundi, Ching Wey David Yav, Buoang Mothobi, Lineo Thahane, Alain Amstutz, Nadine Bachmann, Getrud Joseph Mollel, Moniek Bresser, Tracy Renée Glass, Daniel Henry Paris, Thomas Klimkait, Maja Weisser, Niklaus Daniel Labhardt

**Affiliations:** 1 Swiss Tropical and Public Health Institute, Basel, Switzerland; 2 Molecular Virology, Department of Biomedicine, University of Basel, Basel, Switzerland; 3 University of Basel, Basel, Switzerland; 4 SolidarMed, Partnerships for Health, Maseru, Lesotho; 5 Ifakara Health Institute, Ifakara, Tanzania; 6 Seboche Mission Hospital, Seboche, Lesotho; 7 Baylor College of Medicine Children’s Foundation Lesotho, Maseru, Lesotho; 8 Baylor College of Medicine, Houston, TX USA; 9 Department of Infectious Diseases and Hospital Epidemiology, University Hospital Basel, Basel, Switzerland

**Keywords:** HIV, Genotypic resistance testing, Drug resistance, Randomised clinical trial, Antiretroviral therapy, Treatment failure, Children, Adolescents, Sub-Saharan Africa

## Abstract

**Background:**

Globally, the majority of people living with HIV have no or only limited access to HIV drug resistance testing to guide the selection of antiretroviral drugs. This is of particular concern for children and adolescents, who experience high rates of treatment failure. The GIVE MOVE trial assesses the clinical impact and cost-effectiveness of routinely providing genotypic resistance testing (GRT) to children and adolescents living with HIV who have an unsuppressed viral load (VL) while taking antiretroviral therapy (ART).

**Methods:**

GIVE MOVE is an open-label randomised clinical trial enrolling children and adolescents (≥6 months to <19 years) living with HIV with a VL ≥400 copies/mL (c/mL) while taking first-line ART. Recruitment takes place at sites in Lesotho and Tanzania. Participants are randomised in a 1:1 allocation to a control arm receiving the standard of care (3 sessions of enhanced adherence counselling, a follow-up VL test, continuation of the same regimen upon viral resuppression or empiric selection of a new regimen upon sustained elevated viremia) and an intervention arm (GRT to inform onward treatment). The composite primary endpoint is the occurrence of any one or more of the following events during the 36 weeks of follow-up period: i) death due to any cause; ii) HIV- or ART-related hospital admission of ≥24 h duration; iii) new clinical World Health Organisation stage 4 event (excluding lymph node tuberculosis, stunting, oral or genital herpes simplex infection and oesophageal candidiasis); and iv) no documented VL <50 c/mL at 36 weeks follow-up. Secondary and exploratory endpoints assess additional health-related outcomes, and a nested study will assess the cost-effectiveness of the intervention. Enrolment of a total of 276 participants is planned, with an interim analysis scheduled after the first 138 participants have completed follow-up.

**Discussion:**

This randomised clinical trial will assess if the availability of resistance testing improves clinical outcomes in children and adolescents with elevated viremia while taking ART.

**Trial registration:**

This trial is registered with ClinicalTrials.gov (NCT04233242; registered 18.01.2020). More information: www.givemove.org.

## Background

Almost three million children and adolescents worldwide are living with HIV [1]. Every day, almost 1000 children and adolescents are newly infected and over 300 die from HIV/AIDS-related causes [1]. Eastern and Southern Africa are particularly affected, accounting for 65% of the epidemic in children and adolescents [1]. While substantial progress has been made towards providing antiretroviral therapy (ART) to all people living with HIV, which can suppress viral replication and prevent onward transmission of HIV [2–4], children and adolescents suffer high rates of treatment failure: among those younger than 15 years who receive ART, reported rates of treatment failure in Eastern and Southern Africa range from 10% (Eswatini) to over 50% (Eritrea, Mozambique, South Sudan) [5].

Treatment failure can be caused by non-adherence to therapy, viral drug resistance, or a combination of both, requiring differentiated clinical management. Without resistance testing, healthcare providers cannot definitively determine whether treatment failure is caused by drug resistance, necessitating an urgent switch of drug regimen, or non-adherence, in which case underlying causes must be addressed and unnecessary switching must be avoided to preserve the limited future treatment options.

Access to genotypic resistance testing (GRT) to detect viral drug resistance is lacking in most low-income settings [6]. As national HIV programs in sub-Sahara Africa struggle with limited resources, the question if resistance testing is of real clinical benefit or rather a “nice to have” is important as it impacts resource allocation within programs. The World Health Organisation (WHO) recommends resistance testing only upon confirmed treatment failure on second-line ART and/or protease-inhibitor-based ART, and even then only after a lengthy process of enhanced adherence counselling followed by a confirmatory viral load (VL) test [7].

A recent systematic review on the impact of genotypic and/or phenotypic resistance testing in ART-experienced individuals only found randomised clinical trials published before 2007, all conducted in Europe, the USA, or South America, only two of which included children and/or adolescents [8]. This review reported a potential slight reduction of virologic failure where resistance testing was available, but little or no difference in mortality, CD4 cell count, progression to AIDS, or adverse events. Among three modelling studies on the cost-effectiveness of GRT in southern Africa, published between 2011 and 2014, conclusions differed greatly [9–11].

Three ongoing randomised clinical trials (in addition to the trial presented here) will assess the usefulness of resistance testing in sub-Saharan Africa: the REVAMP study, conducted in South Africa and Uganda, is assessing the feasibility, effectiveness, and cost-effectiveness of GRT upon detection of viremia in adults taking non-nucleoside reverse transcriptase inhibitor- (NNRTI-)based first-line ART [12]. A trial in Tanzania, including all age groups, implements GRT upon confirmation of treatment failure after enhanced adherence counselling [13]. Finally, the Opt4Kids trial assesses the impact of a combination of point-of-care VL testing and targeted resistance testing among children on first-line ART in Kenya [14].

We report here the protocol of the trial: *Genotype-Informed* Versus *Empiric Management Of VirEmia (GIVE MOVE) in HIV-Infected Children and Adolescents on Antiretroviral Therapy: An Open-Label Randomised Clinical Trial*. GIVE MOVE is among the first randomised clinical trials assessing the clinical impact of providing GRT to children and adolescents with viremia while on first-line ART, with key differences in study design compared to the above-mentioned ongoing trials.

## Methods

### Aim

The GIVE MOVE trial assesses whether timely provision of GRT upon detection of viremia improves health outcomes for children and adolescents on first-line ART when compared to the current standard of care. In the case of an observed clinical benefit, the cost-effectiveness of this intervention will be assessed. Combined, these results will provide evidence on whether the availability of GRT should be prioritised for children and adolescents living with HIV in resource-limited settings.

### Design and study setting

GIVE MOVE is a multi-centre, parallel-group (1,1 allocation), open-label, superiority randomised clinical trial conducted in Lesotho and Tanzania. These two countries are home to 21,000 and 150,000 children and adolescents living with HIV [1], respectively, and have a reported adult HIV prevalence of 22.8% in Lesotho and 4.8% in Tanzania [5].

Enrolment will take place at four sites. In Lesotho, these are the Satellite Centres of Excellence of the Baylor College of Medicine Children’s Foundation Lesotho (‘Baylor Clinics’) located in Hlotse, Butha-Buthe, and Mokhotlong. In Tanzania, the study is conducted at the One-Stop Clinic of the Chronic Diseases Clinic Ifakara at Saint Francis Referral Hospital in Ifakara, Kilombero District. In both countries, additional sites have been identified for potential inclusion at a later stage.

GRT takes place at the laboratory of Seboche Mission Hospital in Butha-Buthe district, Lesotho and at the laboratory of the Ifakara Health Institute in Ifakara, Tanzania. All other laboratory diagnostics are conducted at laboratories associated with the respective sites.

### Participants

Potential participants are identified through pre-screening of routine medical records.

Inclusion criteria are: being in care in a study site; age ≥6 months and <19 years; latest HIV VL result ≥400 copies/mL (c/mL); being on a first-line ART regimen (defined as never having had a regimen change due to virologic failure); having been on an unchanged ART regimen for ≥6 months; phlebotomy for the latest VL test done <3 months before screening; and written informed consent.

Exclusion criteria are: an indication for treatment switch according to WHO guidelines at screening; initiation of the first session of enhanced adherence counselling >2 weeks prior to screening; an intention to transfer out of the study site (and not into a different study site) within 3 months after randomisation; already being enrolled in another study if judged as non-compatible by the (Local) Principal Investigator; being pregnant or breastfeeding at screening (no exclusion based on pregnancy or breastfeeding after enrolment); acute illness requiring hospitalisation at screening (no exclusion based on hospitalisation after enrolment); and having received an HIV resistance test in the last 12 months.

### Consent procedures, screening, and randomisation

Consent is provided by the participant if aged ≥16 years (Lesotho) or ≥18 years (Tanzania), and by the caregiver for younger participants. Minors aged ≥6 years additionally receive age-appropriate study information and provide informed assent. Written informed consent and, where applicable, written informed assent is a prerequisite for participation in this study. Details on consenting procedures are listed in the declarations below. Formal screening, including a non-routine pregnancy test for female adolescents aged ≥12 years, takes place only after consent (and assent, if applicable) has been obtained.

Eligible and consenting individuals are enrolled and randomised in a 1:1 ratio to the intervention and control arms. Randomisation is stratified by country (Lesotho or Tanzania), age at enrolment ([≥6 months to <12 years] or [≥12 years to <19 years]), and type of ART regimen at enrolment (NNRTI-, protease inhibitor- (PI-), or integrase strand transfer inhibitor- (INSTI-)based regimen), using permuted blocks with varying block size. Randomisation is automated using the electronic data capture software MACRO version 4.8.1 (Elsevier) once eligibility and consent have been confirmed and entered into the database, thereby maintaining concealment of allocation.

### Control and intervention arm

The control arm is largely based on the WHO and national guidelines [7, 15, 16]: Participants in the control arm receive (at least) three sessions of adherence counselling followed by a second VL test. Onward treatment is determined by the outcome of this second VL test: sustained viremia ≥400 c/mL triggers a switch to a second-line ART regimen selected based on empiric criteria according to national guidelines [15, 16], whereas viral resuppression to <400 c/mL results in continuation of the current regimen.

This cut-off of 400 c/mL for viral suppression was selected based on the growing body of evidence that the cut-off of 1000 c/mL currently recommended by the WHO [7] and the national guidelines of the project countries [15, 16] may be too high [17–21]. While guidelines suggest the confirmatory VL test should be delayed and adherence counselling should continue in the case of ‘ongoing poor adherence’, the GIVE MOVE protocol allows for this only upon evidence of non-adherence defined as i) a pill count <90%, or ii) a self-reported period of no drug intake of ≥2 days during the past 4 weeks.

Participants in the intervention arm receive an intervention package consisting of: i) GRT by Sanger sequencing completed by an in-country laboratory (target turn-around time: 2 weeks); ii) review of the GRT result by at least three members of a GRT Expert Committee, providing a recommendation for onward treatment (target turn-around time: 1 week); iii) GRT-informed choice of onward therapy; and iv) GRT-informed further adherence counselling.

**Fig. 1:**
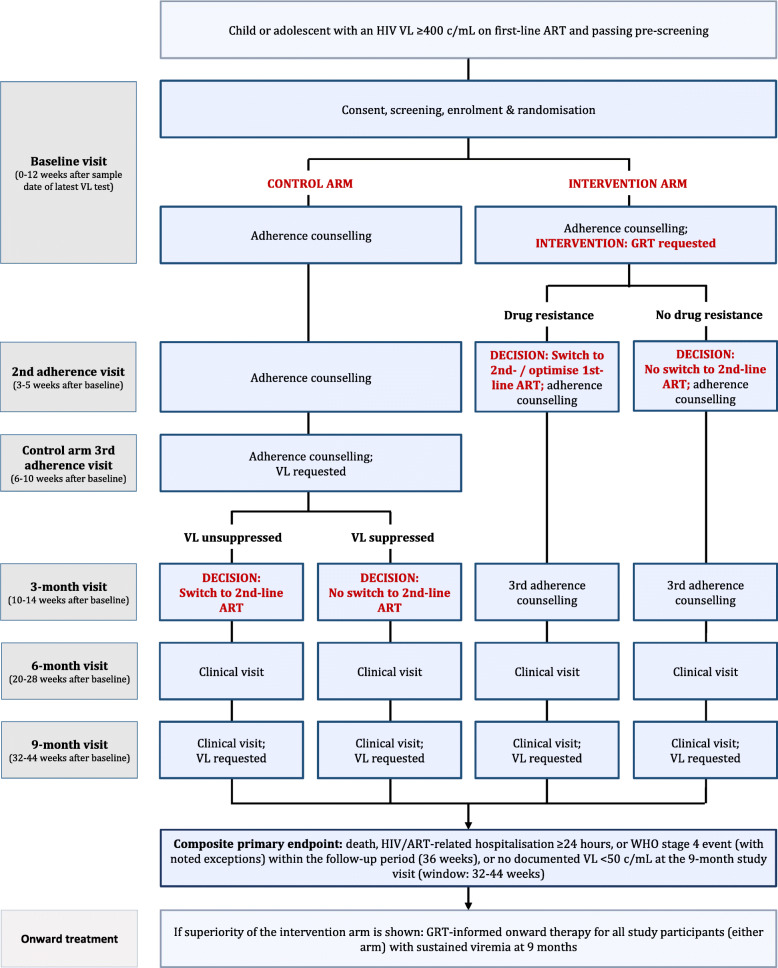
Flow chart of GIVE MOVE treatment algorithm and study visits. Detailed procedures at each study visit are listed in Fig. 2. GRT: genotypic resistance testing; VL: viral load

**Fig. 2:**
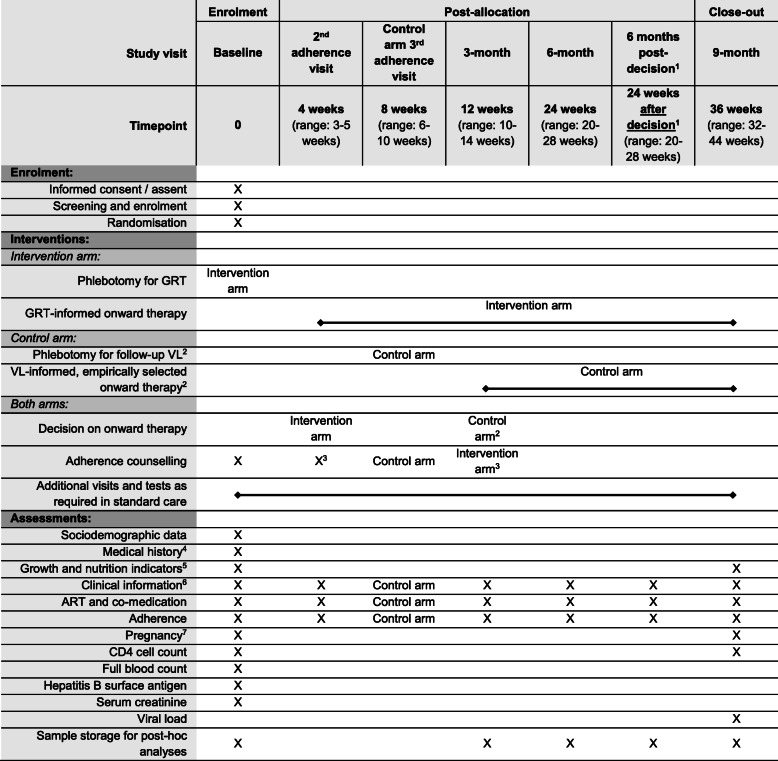
SPIRIT diagram of study procedures. Footnotes: ^1^ Conducted 24 weeks (range: 20–28 weeks) after the visit in which the follow-up viral load result (control arm) or the GRT result (intervention arm) is first available; may coincide with another study visit. ^2^ May be delayed upon evidence of poor adherence, defined as a pill count of <90% and/or a self-reported period of no drug intake of ≥2 days in the last 4 weeks. ^3^ In intervention arm: informed by GRT result. ^4^ Clinical information at ART initiation; previous ART regimens; exposure to prevention to mother-to-child transmission strategies. ^5^ Height, weight, middle upper arm circumference (if aged <5 years), nutritional status. ^6^ WHO stage; co-morbidities; symptoms and side-effects; new hospitalisation. ^7^ Female participants aged ≥12 years. GRT: genotypic resistance testing; VL: viral load

The main study visits for each arm are shown in Fig. 1. Additionally, a ‘6 months post decision visit’ takes place 24 weeks (range: 20—28 weeks) after the decision on onward therapy, i.e. after the visit in which the follow-up VL result (control arm) or the GRT result (intervention arm) become available. Depending on the timing, this ‘6 months post decision visit’ is either combined with another study visit or conducted separately. Any additional visits and laboratory tests taking place according to the standard of care or clinical necessity (including but not limited to: more frequent clinical visits upon pregnancy; check-up visits after modifications to the ART regimen; clinical indication) are recorded. Missing participants will be traced, contacted and encouraged to return back to care. The study procedures at each study visit are shown in the SPIRIT diagram in Fig. 2.

### Endpoints

The composite primary endpoint is the occurrence of any one or more of the events i) death due to any cause during the follow-up period (36 weeks), ii) HIV- or ART-related hospital admission of ≥24 h duration (possibly, probably or definitely related to HIV or ART, judged by the endpoint committee blinded to the study arm) during follow-up, iii) new clinical WHO stage IV event (excluding lymph node tuberculosis, stunting, oral or genital herpes simplex infection and oesophageal candidiasis; judged by the endpoint committee blinded to the study arm) during follow-up, and iv) no documentation of a suppressed VL (<50 c/mL) at 36 weeks follow-up (window: 32–44 weeks).

The secondary and exploratory endpoints are listed in Table 1.

**Table 1:** Secondary and exploratory endpoints

Endpoint	Definition	Timeframe
***Secondary endpoints***		
Death due to any cause	Proportion of participants confirmed dead during the follow-period among all participants enrolled	Within 36 weeks after enrolment
HIV- or ART-related hospitalisation of ≥24 h duration	Proportion of participants with HIV- or ART-related hospital admission(s) of ≥24 h duration (possibly, probably or definitely related to HIV or ART, judged by the endpoint committee blinded to the study arm) during the follow-up period among all participants enrolled	Within 36 weeks after enrolment
New clinical WHO stage 4 event(s)	Proportion of participants with new clinical WHO stage 4 event(s) (excluding lymph node tuberculosis, stunting, oral or genital herpes simplex infection and oesophageal candidiasis, judged by the endpoint committee blinded to the study arm) among all participants enrolled	Within 36 weeks after enrolment
Without documentation of a suppressed VL	Proportion of participants without documentation of a VL <50 c/mL at 9 months among all participants enrolled	32—44 weeks after enrolment
Loss to follow-up	Proportion of participants with no documented clinic visit at 9 months among all participants enrolled	32—44 weeks after enrolment
Observed virologic failure	Proportion of participants with a VL ≥50 c/mL among all participants with a VL result at 9 months	32—44 weeks after enrolment
Composite endpoint at 6 months after the decision on onward treatment	Proportion of participants among all participants enrolled experiencing any one or more of the events i) death due to any cause within 24 weeks of the decision on onward treatment, ii) HIV- or ART-related hospital admission of ≥24 h duration (possibly, probably or definitely related to HIV or ART, judged by the endpoint committee blinded to the study arm) within 24 weeks of the decision on onward treatment, iii) new clinical WHO stage IV event (excluding lymph node tuberculosis, stunting, oral or genital herpes simplex infection and oesophageal candidiasis; judged by the endpoint committee blinded to the study arm) within 24 weeks of the decision on onward treatment, and iv) no documentation of a suppressed VL (<50 c/mL) at 6 months (20–28 weeks) after the choice of onward treatment. The time point of the decision on onward treatment is defined as the first visit in which the follow-up VL result (control arm) or the GRT result (intervention arm) is available.	i-iii): within 24 weeks after the decision on onward therapy; iv): 20—28 weeks after the decision on onward therapy
***Exploratory endpoints***		
Time to documented viral suppression	Time to achieving a VL <50 c/mL; considering VL testing done with samples from the 3-, 6- and 9-month study visits in both arms	Assessed at 3- (10—14 weeks after enrolment), 6- (20—28 weeks after enrolment), and 9-month study visit (32—44 weeks after enrolment)
Drug regimen switches in the absence of major resistance-associated mutations and/or non-switches in the presence of major resistance-associated mutations	Proportion of participants with ART regimen switches in the absence of major resistance-associated mutations and/or non-switches in the presence of major resistance-associated mutations among all participants enrolled (as identified by Sanger sequencing, according to the Stanford HIV drug resistance database).	Assessed at enrolment and at 9-month study visit (32—44 weeks after enrolment)
Proportion with new resistance-associated mutations emerged within the study period	Proportion of participants with new resistance-associated mutations emerged within the study period among all participants enrolled	Change from enrolment to 9-month study visit (32—44 weeks after enrolment)

The first four secondary endpoints are the individual components of the composite primary endpoint.

### Sample size

We hypothesise that 35% of participants in the control arm will reach the primary endpoint. With α = 0.05 and 80% power, a sample size of 276 participants is needed to detect a clinically relevant reduction in the primary endpoint of 15% in the intervention arm.

### Planned analyses

Analysis and reporting will follow CONSORT guidelines [22] and intention-to-treat (ITT) principles including all participants as randomised. In addition, a per-protocol analysis of the primary endpoint will include all randomised participants who completed the study without a protocol violation. A flowchart will describe the inclusion and follow-up of participants by study arm. Baseline characteristics will be described by study arm with summary statistics such as median and interquartile range or number and percentage; no formal testing between arms will be performed [23]. The primary endpoint as well as categorical secondary and exploratory endpoints will be assessed using a logistic regression model, reporting odds ratios and risk differences with standard errors estimated using the delta method [24]. The exploratory endpoint of time until documented viral suppression will be assessed with Cox proportional hazard models, reporting hazard ratios. All estimates will be reported with 95% confidence intervals. All models will be adjusted for the stratification factors of country, age, and ART regimen at enrolment. Subgroup analyses are planned by country (Lesotho or Tanzania), sex (female or male), age ([≥6 months to <12 years] or [≥12 years to <19 years]), and ART regimen at enrolment (NNRTI-, PI-, or INSTI-based regimen).

### Interim analysis

An interim analysis for efficacy and inefficacy is planned once 138 participants (50% of the target number of participants) have completed the 9-month study visit and/or reached the primary endpoint. The trial may be concluded early for success if a significant difference between the study arms is achieved for the composite primary endpoint. We will use the conservative Haybittle-Peto stopping level of *p* = 0.001 [25]. The trial may be stopped for inefficacy if the odds ratio is greater than 1 and the two-sided 95% confidence interval does not contain the alternative hypothesis (i.e., odds ratio of 0.57, [26]). More details are provided in the statistical analysis plan.

The interim analysis will be conducted by an independent statistician. The results will be reviewed by a Data Safety Monitoring Board, who will issue a recommendation to continue or stop the trial to the Steering Committee. For this recommendation, additional information (i.e. new emerging evidence) may also be taken into account. The Steering Committee will vote on and thereby determine the continuation or termination of the trial. In the event of a tie, the Sponsor/Chief Investigator will cast the deciding vote. If the decision is taken to stop the trial, recruitment will be suspended but participants who are already enrolled will continue to be followed for the primary and secondary outcomes.

### Data collection and management

Data is captured online in electronic case report forms in the password-protected MACRO database, which generates an audit trail. On the electronic report forms, participants are identified by a unique identifier and no participant names are stored in the database. A paper-based participant identification list, the Informed Consent/Assent Forms, and paper-based source documents are kept under lock and key at each study site. All study data and documentation will be archived for at least 10 years after completion of the study.

### Plasma collection and storage

Participants will undergo phlebotomy at enrolment and 2 (control arm only), 3, 6 and 9 months after enrolment (see Spirit diagram in Fig. 2). Study-related phlebotomy will be limited to age-appropriate volumes per blood draw, defined as ≤5 mL for participants <5 years; ≤10 mL for participants ≥5 and <10 years; ≤15 mL for participants ≥10 and <15 years; and ≤25 mL for participants ≥15 years [27]. The sites receive training and written guidance on the safe blood volumes in paediatric patients and the prioritisation of laboratory procedures in the event that insufficient blood is available to perform all tests as per protocol.

Biological material is identified by the participant’s study ID and processed or stored at −80 °C at the laboratory site in charge. Consent is collected for further use of samples in future studies, subject to approval from the relevant ethics committee(s).

### Monitoring

In Lesotho, GIVE MOVE is monitored by the Monitoring Group of the Clinical Operations Unit at the Swiss Tropical and Public Health Institute, as well as monitors from SolidarMed Lesotho who are supervised by this group. In Tanzania, the trial is monitored by the Ifakara Health Institute. The first two participants per site and approximately 10% of the total number of participants will undergo 100% source data verification. The remaining participants will undergo source data verification of all key data as defined in the monitoring plan. For each study site a site initiation visit, regular routine monitoring visits and a close-out visit are planned.

In addition, the Baylor Clinics are audited on a half-yearly basis by the Baylor College of Medicine Children’s Foundation Lesotho.

### Ethical considerations

This study has been approved by the relevant ethics committees and, if applicable, other authorities in the project countries. In addition, a Swiss ethics committee provided a statement confirming the trial meet ethical requirements. Details are listed in the declarations below.

All participants/caregivers are informed that participation is voluntary and that they may withdraw from the study at any time. Participants do not receive any form of remuneration, though transport costs to the study site are compensated for participants and up to one caregiver.

Pregnancy (assessed by a pregnancy test in female adolescents aged ≥12 years during screening) or breastfeeding at enrolment are exclusion criteria; however, pregnancy after enrolment does not lead to exclusion. Participants who become pregnant during the study period will receive additional services including more frequent visits and additional laboratory testing in accordance with the national guidelines [15, 16]. If births occur during the study period, the HIV status and health of the new-born will be recorded.

The following serious adverse events will be captured and reported to the ethics committees: any untoward medical occurrence that i) results in death or is life-threatening; ii) requires in-patient hospitalisation or prolongation of existing hospitalisation; iii) results in persistent or significant disability or incapacity; or iv) causes a congenital anomaly or birth defect.

In the case that the intervention proves beneficial, all participants will receive GRT if they still have an elevated VL at study closure.

### Nested study on cost-effectiveness

A nested study will assess the cost and cost-effectiveness of GRT. For this purpose, the number of clinical visits and care received at each visit (e.g. counselling, clinical exam with a doctor/nurse), the number and duration of hospitalisations, concomitant medication, and all requested laboratory tests are recorded for each participant.

### Trial registration

This trial has been registered with ClinicalTrials.gov (NCT04233242; registered 18.01.2020; https://clinicaltrials.gov/ct2/show/NCT04233242). Further information is available on the trial website: www.givemove.org.

## Discussion

Evidence to guide the management of treatment failure in children and adolescents in low-income settings is desperately lacking, endangering the UNAIDS vision of an AIDS-free generation by 2030 [28].

Early, successful ART is key to child development as it reduces mortality and morbidity, improves neurocognitive and growth outcomes [29, 30], and preserves future therapeutic options. However, intention-to-treat analyses report that 20—30% of children and adolescents have an unsuppressed VL 1 year after starting first-line ART [31, 32]. Similarly, a recent systematic review showed that after undergoing enhanced adherence counselling upon detection of viremia, subsequent resuppression was achieved by a little over half (50.4%) of adults, but only 31.2 and 40.4% of children and adolescents, respectively [33]. Even among children whose ART regimen is switched to second-line, resuppression rates remain low [34].

The GIVE MOVE trial assesses the feasibility, clinical impact, and cost-effectiveness of GRT to guide the clinical management of viremia despite first-line ART in children and adolescents. We hypothesise that GRT will substantially improve treatment outcomes by allowing for differentiated care customised according to the individual child/adolescent’s health situation and needs, i.e. targeted adherence support for those without drug resistance and a rapid switch to an optimised ART regimen (with potential additional adherence support) in those with drug-resistant HIV, as well as by reducing the time to appropriate clinical action.

This trial has several limitations. Given the nature of the intervention, blinding of participants or healthcare professionals is not possible. Furthermore, the trial cannot make full use of the potential of GRT to reduce time to clinical decision-making: due to the ethical necessity of consenting participants before conducting any non-routine procedures, enrolment and phlebotomy for GRT take place ideally at the first clinic visit after a routine VL test (generally after 1 month). In clinical practice, however, it would be possible to use blood remaining after VL testing to immediately conduct GRT if viremia is detected, and provide both the VL and the GRT result at the subsequent clinic visit. Thus, the GIVE MOVE trial will likely underestimate the potential benefit of reducing time to clinical decision-making.

However, this trial also has several strengths. The multi-site approach, as well as the fact that this pragmatic trial relies heavily on existing infrastructure at the study sites and the logistical capacities of the in-country partners, will increase external validity. The inclusion of participants on newer INSTI-based ART regimens (notably dolutegravir-based regimens) ensures that results will remain relevant for years to come as dolutegravir-based ART becomes increasingly available [35], and the focus on children and adolescents ensures that the needs of particularly vulnerable age groups are addressed.

In conclusion, the GIVE MOVE trial will assess if the availability of GRT for children and adolescents with unsuppressed VLs while taking ART improves clinical outcomes and if it is cost-effective. While funding for national HIV programs in Africa is stagnating or even decreasing, it is more important than ever that resource allocation gives highest priority to evidence-based interventions. Data from GIVE MOVE will provide evidence to program managers and policymakers for the decision on whether access to GRT is an intervention to which further resources should be allocated.

### Trial status

The trial was launched at the first site (Baylor Clinic Hlotse) on 20.02.2020, and the first participant was enrolled on 03.03.2020. As per 06.10.2020, all sites are following study protocol v1.3 (dated 27.02.2020), 33 participants have been enrolled, and all four sites have enrolled at least one participant. Enrolment is expected to continue until mid- to late 2021, with a subsequent follow-up period of up to 11 months.

## Data Availability

Results of this research will be disseminated at the district and/or national level to stakeholders within the project countries, as well as at the international level through peer-reviewed publications and academic conferences. Upon publication of the trial results, a subset of the key pseudo-anonymised individual participant data collected during the study, along with a data dictionary, will be made available through the data repository Zenodo. Case report forms and other key study documents will also be made available upon publication of results. The full dataset will be made available upon request to the Department of Medicine at the Swiss Tropical and Public Health Institute and after signing a data confidentiality agreement.

## Abbreviations

ART: Antiretroviral therapy
c/mL: Copies per millilitre
GIVE MOVE: Genotype-Informed Versus Empiric Management Of VirEmia
GRT: Genotypic resistance testing
HIV: Human immunodeficiency virus
INSTI: Integrase strand transfer inhibitor
ITT: Intention-to-treat
NNRTI: Non-nucleoside reverse transcriptase inhibitor
PI: Protease inhibitor
VL: Viral load
WHO: World Health Organisation
